# The whistle-smile reflex: a missed sign

**DOI:** 10.1055/s-0042-1758396

**Published:** 2022-12-19

**Authors:** Mateus Damiani Monteiro, Giovanna Testa Brustolin, Eurípedes Gomes de Carvalho Neto, Carlos Roberto de Melo Rieder

**Affiliations:** 1 Santa Casa de Misericórdia de Porto Alegre, Departamento de Neurologia, Porto Alegre RS, Brazil.

**Keywords:** Parkinson Disease, Physical Examination, Doença de Parkinson, Exame Físico

## Abstract

In this paper, we present a historical review of the whistle-smile reflex, a semiological sign missed in the literature and clinical practice.

## Resumo

Apresentamos neste artigo uma revisão histórica do reflexo do assovio-sorriso, um sinal semiológico esquecido na literatura e na prática clínica.

## INTRODUCTION



**Video 1** The whistle-smile reflex. The patient and the healthy person gave us written consent to use their images. https://www.arquivosdeneuropsiquiatria.org/wp-content/uploads/2022/08/ANP-2022.0120-Video.mov


**Figure 1:**
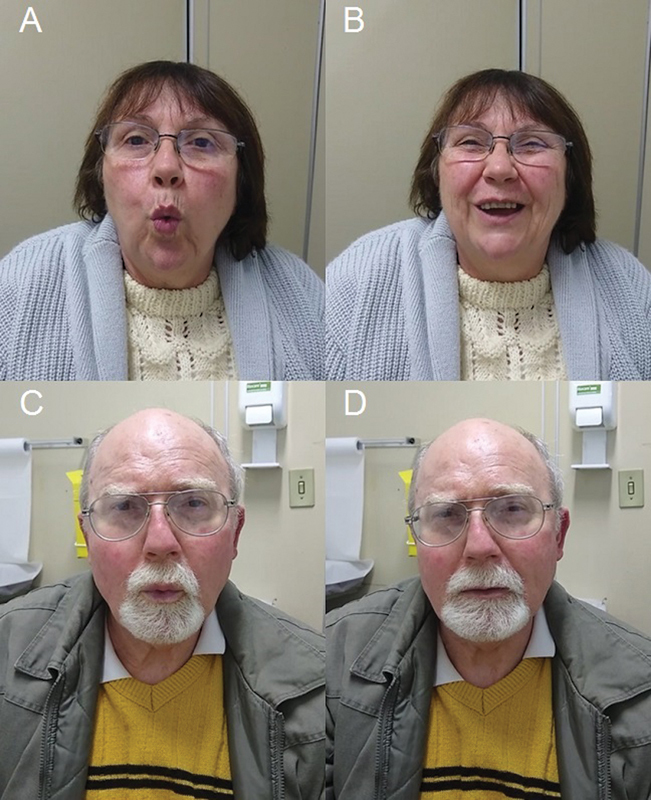
Non-Parkinson disease patient's smiles after whistling (A-B). Parkinson disease patient shows no reaction after doing the same (C–D).

In 1943, Frederic Hanes described the whistle-smile reflex, also known as Hanes sign: when asked to whistle, a healthy person does so and then smiles after the unusual physician's request. Otherwise, a patient with parkinsonism performs the whistle but does not smile after whistling due to the facial bradykinetic movement 1 ( Video 1 and Figure 1 ).

Dr. Hanes was a neurologist and professor of medicine at Duke University. During his many years in Durham, North Carolina, he explored the presence of hypomimia as a semiological tool for the diagnosis of Parkinson disease (PD); finding, during his evaluations, what he considers a helpful and reliable sign.

Lack of facial expression is a well-known feature of parkinsonian syndrome and an essential clue to diagnosing PD. Although James Parkinson did not mention hypomimia in his seminal work “An Essay on the Shaking Palsy” 2 , it was recognized as an important semiological finding in a patient with PD in early works by authors such as Charcot, Gowers, and Wilson. 3 The facial movement impairment may be not only voluntary but also spontaneous and emotional. The involvement of emotional expressions can be noticed with spontaneous smiling, reflected in reduced frequency and opening degree of the mouth. 4

The whistle-smile reflex, alongside the Rolex sign 5 and the floating door sign, 6 lost its role in assessing a parkinsonian patient throughout the years. Although it is an interesting possibility to use the whistle-smile reflex in clinical practice, this reflex has not been revised in detail over the years, and we still do not have established its sensitivity and specificity. Therefore, it deserves to be remembered, not only for its historical importance but for its possible semiological value in clinical practice, and further studies are needed for it to become a routine tool in the assessment of a parkinsonian patient.
